# Osteosarcoma cell proliferation and survival requires mGluR5 receptor activity and is blocked by Riluzole

**DOI:** 10.1371/journal.pone.0171256

**Published:** 2017-02-23

**Authors:** Sally Liao, Yuleisy Ruiz, Hira Gulzar, Zarina Yelskaya, Lyes Ait Taouit, Murielle Houssou, Trisha Jaikaran, Yuriy Schvarts, Kristina Kozlitina, Upal Basu-Roy, Alka Mansukhani, Shahana S. Mahajan

**Affiliations:** 1 Department of Medical Laboratory Sciences, Hunter College, City University of New York, New York, NY, United States of America; 2 Department of Microbiology & Perlmutter Cancer Center, NYU Langone Medical Center, New York, NY, United States of America; 3 Brain and Mind Research Institute, Weil Cornell Medical College, New York, NY, United States of America; Rutgers University, UNITED STATES

## Abstract

Osteosarcomas are malignant tumors of bone, most commonly seen in children and adolescents. Despite advances in modern medicine, the poor survival rate of metastatic osteosarcoma has not improved in two decades. In the present study we have investigated the effect of Riluzole on a human and mouse metastatic osteosarcoma cells. We show that LM7 cells secrete glutamate in the media and that mGluR5 receptors are required for the proliferation of LM7 cells. Riluzole, which is known to inhibit glutamate release, inhibits proliferation, induces apoptosis and prevents migration of LM7 cells. This is also seen with Fenobam, a specific blocker of mGluR5. We also show that Riluzole alters the phosphorylation status of AKT/P70 S6 kinase, ERK1/2 and JNK1/2. Thus Riluzole is an effective drug to inhibit proliferation and survival of osteosarcoma cells and has therapeutic potential for the treatment of osteosarcoma exhibiting autocrine glutamate signaling.

## Introduction

Osteosarcoma is the most common malignant primary cancer of bone occurring in young adults and adolescents [1, 2]. The 5-year survival rate has not improved in the last two decades [3]. Thus, new therapeutic strategies need to be developed in order to improve the treatment and survival outcomes in osteosarcoma patients.

Glutamate is a major excitatory neurotransmitter in the human central nervous system, playing an important role in memory and learning processes. It also plays a key role in cellular homeostasis and serves as a fuel for metabolic pathways in other tissue types [4, 5]. Recently the role of glutamate signaling has been discovered in peripheral tissues including bone, playing a vital role in bone survival and differentiation [6–9]. Importantly, some cancers have been shown to gain growth advantage by exploiting autocrine/paracrine glutamate signaling [10–12]. Non-neuronal cancers such as breast cancer, melanoma, and prostate cancer [11–15] utilize glutamaterigic system for their growth by over expression of glutamate receptors [11, 12]. Furthermore, cancer types such as rhabdomyosarcoma, neuroblastoma, thyroid carcinoma, lung carcinoma, astrocytoma, multiple myeloma, lung carcinoma, colon adenocarcinoma, T cell leukemia cells, breast carcinoma, colon adenocarcinoma including brain tumor cells, also express glutamate receptors suggesting that glutamate might play a role in these cancers [16].

There are two types of glutamate receptors, ionotropic and metabotropic receptors. Ionotropic glutamate receptors are ion channels such as NMDA, AMPA and Kainate receptors. Metabotropic glutamate receptors, mGluR, are G protein coupled receptors and are categorized into Group I, Group II and Group III receptors depending upon the homology, agonist selectivity and signal transduction pathways. Both ionotropic and metabotropic glutamate receptors are expressed in the brain and peripheral tissues [17–19]. It is known that some iontotropic and metabotropic glutamate receptors are aberrantly expressed in several types of cancers [11, 20]. In this context, exogenous expression of metabotropic glutamate receptor 1 in immortalized primary baby mouse kidney cells induced tumorigenicity [21]. Although glutamate receptors are normally expressed in brain, several gliomas utilize the glutamatergic system for the progression of malignancy [22, 23]. Furthermore, triple negative breast cancer cell lines, which lack estrogen receptor (ER), progesterone receptor (PR) and human epidermal growth factor receptor (HER2/neu), express metabotropic glutamate receptor 1, mGluR1. Treatment of such triple negative breast cancer with pharmacological agents like Riluzole, a glutamate release inhibitor, inhibits cell proliferation [24]. Interestingly, Riluzole has been shown to prevent proliferation of glioblastoma cells, U87, in culture and in xenograft models [25, 26]. Moreover, Riluzole has been observed to reduce the growth of cancer cells in culture or in xenograft models for melanoma, breast and prostate cancers [24, 27, 28]. Riluzole in a clinical trial for melanoma patients proved to be very promising and showed decreased tumor size or decrease intensity on PET scan in significant number of patients that were enrolled in this study [29]. Another study using melanoma cell lines and xenograft showed that Riluzole is more effective when used in combination with mTOR inhibitor [30].

Based on current literature and the therapeutic promise of Riluzole in some cancers, we have investigated Riluzole as a potential therapeutic agent for osteosarcoma, using LM7 cells. LM7 cells are human metastatic osteosarcoma cells that show aggressive and invasive growth behavior [31]. Towards this aim, we have investigated the role of glutamate in survival, proliferation and migration of LM7 cells. Our results demonstrate that Riluzole blocks proliferation, induces apoptosis and prevents migration of LM7 cells. Furthermore, Riluzole treatment inhibits glutamate signaling through PI3K/AKT/mTOR and other pathways in LM7 proliferation. Importantly, knockdown of mGluR5 prevents cell proliferation in LM7 cells. These data demonstrate the importance of mGluR5 signaling in osteosarcoma growth and provide support for Riluzole as a potential drug for treating osteosarcoma.

## Materials and methods

### Cell culture

Human osteosarcoma, LM7 cells [31] and mouse osteosarcoma cells [32, 33] were maintained in DMEM supplemented with 4.5% glucose, 1mM pyruvate, 2mM glutamate, 10% fetal bovine serum, 100 units/mL penicillin and 100 μg/mL streptomycin. Cells were passaged every 4 days. Cells were maintained at 37°C with 95% air and 5% CO_2_. When indicated, cells were seeded in DMEM media without glutamate, penicillin and streptomycin and 0.5% fetal bovine serum.

### Glutamate assay

Glutamate assay was carried out using a glutamate assay kit from BioVision Incorporated as per the manufacturer’s instructions. Briefly, 500 cells were seeded in 24 well plates in 1 ml of media devoid of glutamine and supplemented with 2mM Glutagro to prevent interference during glutamate assay. On day 0, cells were seeded, the cell viability was determined and the media was collected for the glutamate assay. Triplicate media samples from independent wells were collected each day until day 7 and cell viability was performed using Trypan blue assay.

### Drug treatments

1.5 X 10^4^ cells were seeded onto polylysine coated coverslips in 24 well plates. Twenty-four hour later the cells were treated with drugs such as 100 μM Riluzole, or 50 μM DHPG (*S*)-3,5-Dihydroxyphenylglycine or 40 μM CPCCOEt ((−)-ethyl (7E)-7-hydroxyimino-1,7a-dihydrocyclopropa[b]chromene-1a-carboxylate) or 300 μM Fenobam as specified in each experiment for 72 hours. All drugs were purchased from R&D systems, Minneapolis, MN.

### Proliferation assay

Proliferation assays were performed as described by Yelskaya et al [25]. Post drug treatment, the cells were fixed in 4% paraformaldehyde for 10 min and permeabilized in 0.2% triton X-100 for 5 min. The cells were washed well with 1X Phosphate buffered saline (PBS) and then blocked with 10% bovine serum albumin made in 1X PBS. Immunocytochemistry was carried out using primary antibodies such as polyclonal rabbit anti-Ki-67 (Thermo Fisher Scientific) followed by Alexa 488-conjugated anti-rabbit secondary antibody. The samples were mounted in mounting media (Molecular Probes) containing DAPI for nuclear staining. Images were captured at 20X magnification using a Zeiss fluorescence microscope. The images were used for counting DAPI positive and Ki-67 positive cells. At least five images were used from each sample and the experiments were repeated at least four times.

### TUNEL assay

The TUNEL assay was performed using the *In situ* cell death detection kit from Roche as per manufacturer’s instructions. Briefly, after cells were fixed with 4% paraformaldehyde and permeabilized in 0.2% Triton X-100, the TUNEL assay was carried by incubating the fixed cells with the TUNEL reagent containing TMR red labeled nucleotides at 37° C for 1 hour. The samples were washed in 1X PBS and mounted in mounting media containing DAPI. Fluorescent images were captured using a Zeiss fluorescence microscope at 20X magnification. The total number of DAPI positive cells and total number of TUNEL positive cells were counted from at least five images from each sample. Each experiment was repeated four times.

### Scratch assay

The scratch assay was carried out as described by Goldberg and Kloog [34]. Briefly, 1 million cells/well were seeded on poly-lysine coated 6-well dishes. After 24 hours media was replaced with glutamate free low serum media (0.5% FBS) containing drugs. After 24 hours, three scratches were made and the media was replaced with fresh media containing drugs. Images were captured at 10X with a phase contrast inverted microscope at 0, 8 and 24 hours. Each sample had three scratches and the experiment was repeated three times. Gap width was calculated by taking 150 measurements from each scratch, using SPOT software (SPOT Imaging), to determine average width. Distance travelled by the cells into the gap was calculated as arbitrary units using the width difference in samples at 0, 8 and 24 hours post scratch.

### Western blotting

Western blotting was carried out using either whole cell extracts solubilzed in 2X SDS loading buffer or Medium salt extraction buffer (250 mM KCl, 10 mM Tris-HCl [pH 7.9], 5% glycerol, 0.25% NP-40, 0.2 mM EDTA, 0.5 mM phenylmethylsulfonyl fluoride, 0.2 mM sodium vanadate, 50 μM sodium fluoride, 1 mM dithiothreitol). Antibodies used were anti total AKT (Cell Signaling Technology) or rabbit anti Phospho-Akt (Ser473) Antibody (Cell Signaling Technology) or rabbit anti phospho T308AKT (Millipore), anti Phospho-p44/42 MAPK (Erk1/2) (Thr202/Tyr204) (D13.14.4E) XP rabbit mAb (Cell Signaling Technology), anti Phospho-p70 S6 Kinase (Thr389) antibody (Cell Signaling Technology), anti p70 S6 Kinase Antibody (Cell Signaling Technology), anti p44/42 MAPK (Erk1/2) Antibody (Cell Signaling Technology) anti Phospho-SAPK/JNK (Thr183/Tyr185) (81E11), rabbit mAb and anti JNK2 (56G8), rabbit mAb (Cell Signaling Technology). We used Anti-metabotropic Glutamate Receptor 5 Antibody (Millipore Sigma) to check expression of mGluR5 and anti mGluR1 (BD Biosciences) for mGluR1 expression. μ-Tubulin (Thermo Fisher Scientific) was used for loading control. Signal was detected using chemiluminiscence using Pierce reagents. Western blots were quantified by measuring the relative densities of the bands using Li-Cor software. The ratio of the phosphorylated protein to total protein was calculated and analyzed by Student’s T test.

### Knock down of mGluR5 expression in LM7 cells

Metabotropic glutamate receptor 5 expression was knocked down using Lentivirus vector TRC version 2, pLKO.1-puro vector, expressing ShRNA for mGluR5 (TRCN0000367851-CCGGTTGTGATCAACGCCATCTATTCTCGAGAATAGATGGCGTTGATCACAATTTTTG). Non-target ShRNA (SCH002) was used as control. The Lentiviruses for mGluR5 and control were produced using 293T cells. LM7 cells were infected with various titers of viruses expressing either ShRNA for mGluR5 or virus containing control ShRNA. Stable cells were selected in media containing puromycin (1.5 μg/ml). Stable cells containing the control ShRNA or expressing ShRNA for mGluR were isolated as individual colonies and tested for expression of mGluR5.

### Colonogenic assay

The colonogenic assay was performed as described by Franken et al [35]. Briefly, 500 cells of mGluR KO or vector control were seeded in puromycin containing media in 6 cm dishes. WT LM7 cells were seeded in media without puromycin in 6 cm dishes. The cells were incubated for about 2 weeks until colonies were visible in WT and control. ShRNA The colonies were fixed in 4% paraformaldehyde and stained with 0.5% Cresyl violet in water. Three independent experiments were performed and the average number of colonies was obtained.

### Statistical analysis

The overall significance was determined by two-way ANOVA using Prism (Graphpad) software and significance between each group was determined by pos-hoc analysis using Bornferoni test.

## Results

### LM7 cells secrete glutamate in the media

**Fig 1:**
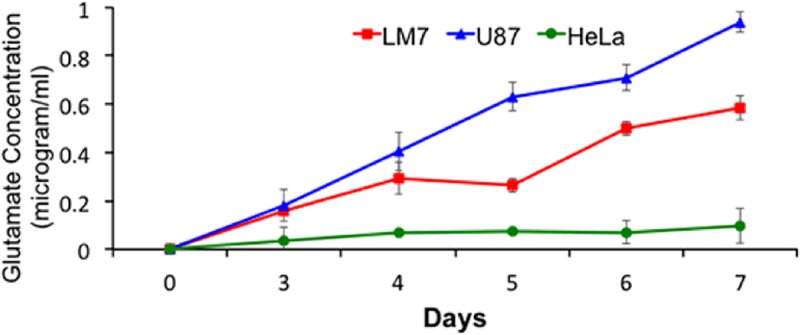
LM7 cells secrete glutamate. Glutamate secreted by LM7 cells was measured in the media from day 0 to day 7. U87 cells were used a positive control represented by triangles and HeLa cells were used as a negative control, shown as circles. LM7 cells are shown as squares. The experiment was repeated three times and the average concentrations are shown.

To determine whether LM7 cells secrete glutamate in the media we assayed glutamate released in the media using a colorimetric glutamate assay as described in the Material and Methods. The results are shown in Fig 1 in which HeLa cells were used as a negative control and show a baseline amount of glutamate in the media. U87, glioblastoma cells, which are known to secrete high levels of glutamate were used as a positive control and showed increasing amount of glutamate secretion increasing from 0.18 μ/ml to above 0.94 μ/ml from day 3 to day 7. Importantly, LM7 cells also secreted glutamate that increased from 0.15 μ/ml to 0.6μ/ml on day 7. These data show that LM7 cells secrete significant levels of glutamate into the media. Cell viability for all cell lines was measured throughout the assay period on each day using Trypan blue assay (Table 1). The cell viability in all samples remained between 98–99% ensuring that the measured glutamate was released into the media by living cells and not from dead or damaged cells. Three independent experiments were performed and the data shown is the average of all three experiments.

**Table 1:** Cell Viability.

Cell Type	Day 1	Day 2	Day 3	Day 4	Day 5	Day 6	Day 7
U87	99.8%+/-0.14	99.7%+/-0.11	99.5%+/-0.10	99.6%+/-0.17	99.6%+/-0.15	99.4%+/-0.17	98.8%+/-0.18
LM7	99.7%+/-0.12	99.7%+/-0.15	99.6%+/-0.12	99.5%+/-0.14	99.6%+/-0.18	99.3%+/-0.18	98.7%+/-0.19
HeLa	99.8%+/-0.07	99.6%+/-0.11	99.7%+/-0.13	99.6%+/-0.16	99.5%+/-0.17	99.4%+/-0.18	98.6%+/-0.20

The percentage of viability for each sample in Fig 1 for all cell lines was determined every day using Trypan Blue. The experiment was repeated three times and the average of three experiments is shown.

### Riluzole inhibits cell proliferation of LM7 and OS482 cells in a dose-dependent manner

**Fig 2:**
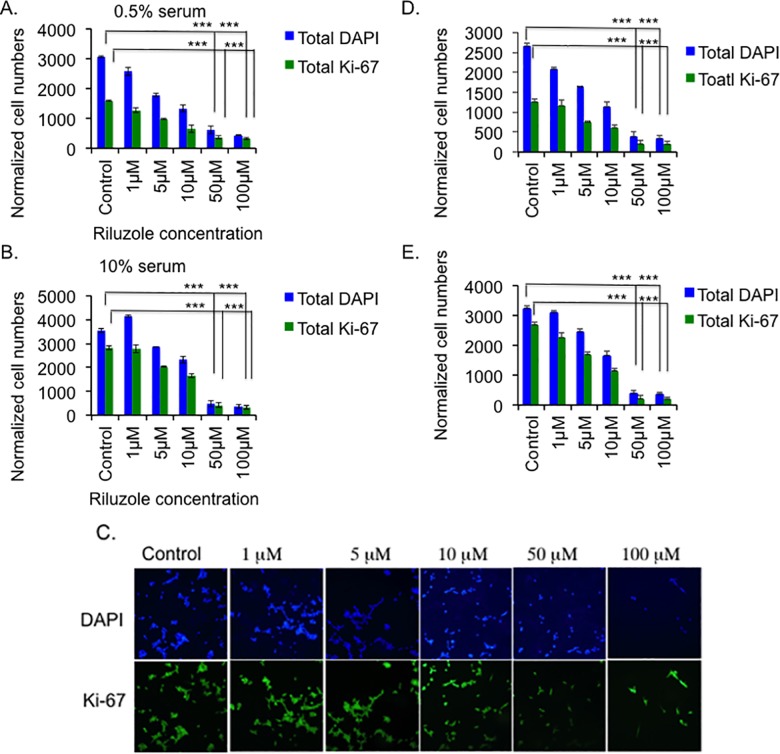
Riluzole inhibits growth of LM7 and OS482 cells both in 0.5% and 10% serum. **A.** Proliferation of LM7 cells was measured in the presence of 0.5% serum containing media. Riluzole was added at various concentrations as indicated for 72 hours. The blue bars represent total number of DAPI positive cells and the green bars represent total number of Ki-67 positive cells (P value <0.001 = ***). **B.** Proliferation of LM7 cells was measured in the presence of 10% serum containing media. Riluzole was added at various concentrations as indicated. The blue bars represent total number of DAPI positive cells and the green bars represent total number of Ki-67 positive cells (P value <0.001 = ***). **C**. Representative images of cells from each sample in 10% serum are shown. **D.** Proliferation of mouse osteosarcoma cells, OS482, was measured in the presence of 0.5% serum containing media. Riluzole was added at various concentrations as indicated for 72 hours. The blue bars represent total number of DAPI positive cells and the green bars represent total number of Ki-67 positive cells (P value <0.001 = ***). **E**. Proliferation of OS482 cells was measured in the presence of 10% serum containing media. Riluzole was added at various concentrations as indicated. The blue bars represent total number of DAPI positive cells and the green bars represent total number of Ki-67 positive cells (P value <0.001 = ***).

To study the effect of glutamate on growth of osteosarcoma cells, we treated LM7 cells with Riluzole. We grew LM7 cells in media containing either 0.5% or 10% serum and treated them with or DMSO as a control or various doses of Riluzole, as indicated. We performed a proliferation assay as described in Materials and Methods. In the presence of 0.5% serum, Riluzole decreased the total number of cells, shown by DAPI positive cells, as the dose of Riluzole increased from 1 μM to 100 μM (Fig 2) (p value <0.001 for 50 μM and 100 μM compared to control). The number of Ki-67 positive proliferating cells also declined with increasing dose of Riluzole (p value <0.001 for 50 μM and 100 μM). In control cells, almost 50% of cells proliferate as indicated by Ki-67 staining (Fig 2A). Interestingly, in the presence of 10% serum, Riluzole at 1 μM slightly increased the total number of cells, indicated by DAPI staining, compared to the control sample. However, the total number of cells declined with higher doses of Riluzole from 5 μM to 100 μM (p value <0.001 for 50 μM and 100 μM). Riluzole also decreased the number of proliferating Ki-67 positive cells in a dose dependent manner from 5 μM to 100 μM (Fig 2B) (p value <0.001 for 50 μM and 100 μM). Representative images in Fig 2C show decreasing number of DAPI positive and Ki-67 positive cells as the dosage of Riluzole increases in 10% serum. As expected, in the presence of 10% serum, control samples showed higher number of proliferating Ki-67 positive cells compared to control sample in 0.5% serum. Furthermore at 0.5% serum, 10 μM Riluzole decreased cell numbers by 50% while in 10% serum, 68% of cells remained compared to the control sample. Importantly, at 50 μM and above, Riluzole decreased total cell numbers to below 20 and 15% in 0.5% and 10% serum, respectively. Similarly, Riluzole inhibited proliferation of mouse osteosarcoma cells, OS482 cells, in a dose dependent manner in 0.5% serum and 10% serum (Fig 2D and 2E) (p value <0.001 for 50 μM and 100 μM). As observed with LM7 cells, OS482 cells showed higher proliferation in 10% serum compared to 0.5% serum (Fig 2D and 2E). Therefore we have utilized Riluzole at 50 μM to treat LM7 and OS482 cells growing in 10% serum containing media for rest of the experiments unless specified. The results in these experiments demonstrate that Riluzole decreases both total numbers of DAPI positive cells and proliferating LM7 cells and OS482 cells. Interestingly, the percentage of proliferating cells in each sample at any given dosage was similar to that of control, which was calculated based on the number of DAPI cells in each sample. The % of proliferation was between 79 to 82 compared to the DAPI cells in 10% serum and did not decrease with the increase in the dosage of Riluzole. This suggested that the proportion of proliferating cells in each sample was same irrespective of the drug treatment or dosage of the drug.

### Riluzole induces apoptosis in LM7 and OS482 cells

**Fig 3:**
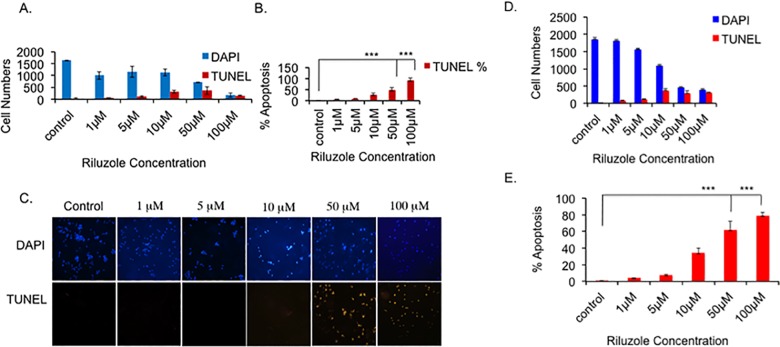
Riluzole induces apoptosis in LM7 and OS482 cells. LM7 cells were seeded and treated with Riluzole at various concentrations or DMSO for control for 72 hours. The cells were fixed and the TUNEL assay was performed. Images were captured and analyzed. **A.** Blue bars represent DAPI positive cells and red bars represent TUNEL positive cells. **B.** Percentage of TUNEL positive cells in each sample was calculated based in total number of DAPI positive cells for that sample (P value <0.001 = ***). **C.** Representative images of the TUNEL assay. **D.** OS482 were treated with various concentrations of Riluzole or DMSO for control and TUNEL assay was performed as in A. **E** Percentage of TUNEL positive cells in each sample was calculated for OS482 cells based in total number of DAPI positive cells for that sample (P value <0.001 = ***).

Our data indicate that treatment with Riluzole decreases LM7 and OS482 cells and the cell numbers decline significantly. The percentage of proliferating cells in all treated samples was similar to that of control and does not decrease with increase in dose of Riluzole. This suggested that the cells may be lost due to cell death. To test whether Riluzole treatment induces cell death by apoptosis, we performed the TUNEL assay. Total number of cells were scored by DAPI staining and apoptotic cells were scored. With increasing doses of Riluzole the number of apoptotic cells increased in LM7 cells and the total number of cells, as shown by DAPI staining, decreased (Fig 3A). Moreover, the percentage of apoptotic cells in the samples increased in a dose dependent manner (Fig 3B and 3C) (p value <0.001 for 50 μM and 100 μM) compared to untreated sample. Similarly, Riluzole increased the number of apoptotic cells in OS482 cells (Fig 3D). Furthermore, the percentage of apoptotic cells increased in a dose dependent manner (Fig 3E) (p value p value <0.001 for 50 μM and 100 μM) compared to untreated control.

### Riluzole blocks migration of LM7 cells

**Fig 4:**
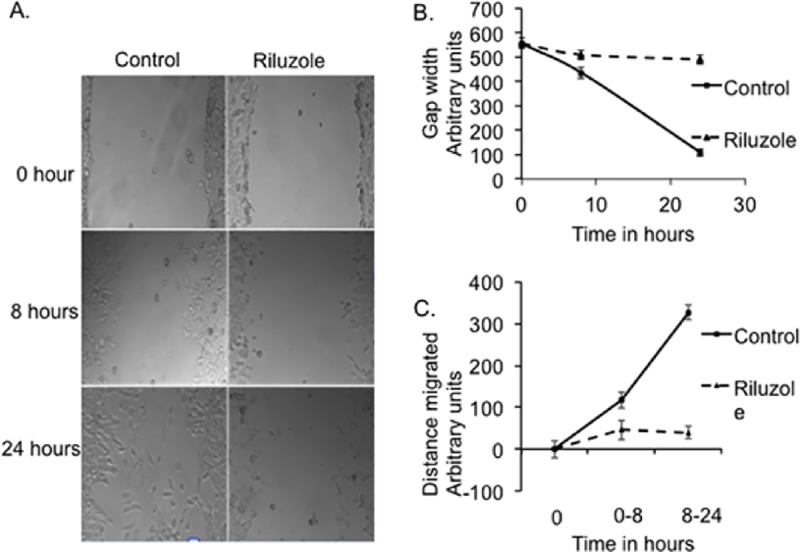
Riluzole prevents migration of LM7 cells. Scratch assay was performed to measure migration of LM7 cells. Cells were either treated with vehicle for control or treated with 100 μM Riluzole, scratched and images of the gap created were captured at 0 time, 8 and 24 hours using phase contrast inverted scope. **A.** Representative images of the scratch in samples at 0 h and 24 hr. **B.** The gap distance was measured from 3 scratches in each image at 0, 8 and 24 hours. **C.** The distance travelled by the cells in the images was measured at 8 and 24 hrs.

We further evaluated the effect of Riluzole in LM7 cell migration using a scratch assay as described in Materials and Methods. Cell migration was determined by measuring the scratch gap distance in samples treated with DMSO or Riluzole. Representative images of control samples and Riluzole treated samples used for measuring the gap width and calculating the distance migrated by cells at 0 hour, 8 hours and 24 hours are shown (Fig 4A). LM7 cells in untreated sample decreased the gap in 24 hours from 553 to 109 arbitrary units. However, Riluzole treated LM7 cells decreased the gap in 24 hours 556 to 490 arbitrary units (Fig 4B). The distance migrated by control cells was 117 and 327 arbitrary units at 8 hours and 24 hours respectively. However, in Riluzole treated samples the cells barely migrated 40 arbitrary units even after 24 hours (Fig 4C). The data from the scratch assay demonstrate that Riluzole prevented migration of LM7 cells.

### Blocking specific metabotropic receptors inhibits LM7 cell growth

**Fig 5:**
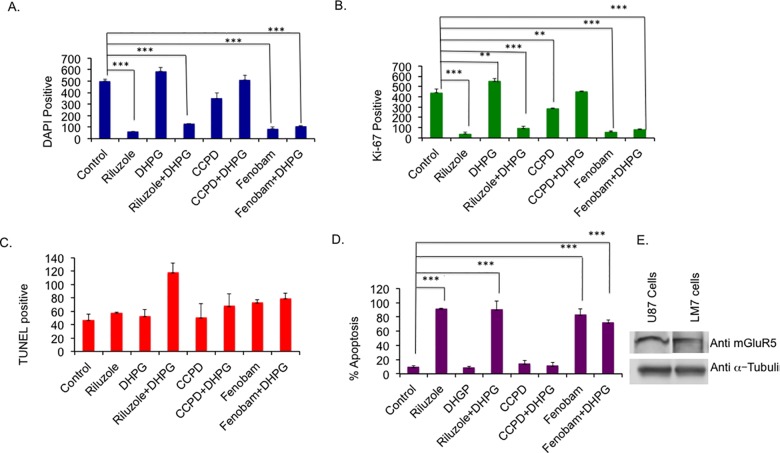
Blocking metabotropic receptors inhibits LM7 growth. **A.** Specific inhibitor of mGluR1, CPCCOEt (CCPD) 40 μM or mGluR5 inhibitor, Fenobam 300 μM, were used to measure total number of cell by DAPI staining. **B.** Proliferation was measured using Ki-67 staining after blocking with specific mGluR inhibitor. **C.** Apoptosis was performed using TUNEL in LM7 cells in the presence of specific mGluR1 or mGluR5 inhibitors. **D.** Percent apoptosis was calculated based on DAPI numbers for samples in Fig 5A. **E.** LM7 cells express mGluR5. Whole cell extracts from LM7 cells were analyzed by Western blot using mGluR5 antibodies. P value <0.01 = **, P value < 0.001 = ****.

To identify the specific type of glutamate receptor involved in LM7 cell growth, we employed specific inhibitors to block mGluR1 and mGluR5 activity in LM7 cells. Cell survival and proliferation were measured in the presence of very potent and specific non-competitive mGluR1 inhibitor, CPCCOEt (40 μM) or negative allosteric modulator of mGluR5, Fenobam (300 μM) (Fig 5A and 5B). Treatment with DHPG (50 μM), a group I metabotropic receptor (mGluR1 and mGluR5) agonist, increased the total number cells as assessed by DAPI staining, and the increase in Ki-67 positive cells over that of control sample was highly significant (p value of 0.01) (Fig 5B). Interestingly, CPCCOEt decreased the cell number, which was not significant but the decrease in Ki-67 positive cell numbers was significant with a p value of 0.01. Samples treated with both CPCCOEt plus DHPG showed a slight increase in the number of DAPI positive and Ki-67 positive cells. However, Fenobam significantly reduced both the DAPI positive and Ki-67 positive cell numbers with a p value of <0.001 (Fig 5A and 5B). Importantly, the inhibition by Fenobam was comparable to that of Riluzole. DHPG treatment did not rescue the inhibition by Fenobam when DHPG was used in combination with Fenobam (P value <0.001). Apoptosis was also measured by TUNEL assay (Fig 5C). The only sample that shows a significant difference in apoptosis compared to the control is the Riluzole plus DHPG treated sample. However, there was a significant decline in the total number of the cells (DAPI positive, Fig 5A) for drug treated samples, due to loss of cells by apoptosis, compared to the untreated sample, therefore we calculated percentage of apoptotic cells from Fig 5A taking DAPI positive cells as 100% for each sample as shown in Fig 5D. Only Riluzole (p value <0.001) and Fenobam (p value <0.001) treatment resulted in the highest percentage of apoptotic cells and DHPG did not rescue the effect of Riluzole when added along with Riluzole (p value <0.001). These results indicate Riluzole and Fenobam are inducing apoptosis most effectively. We further tested the expression of mGluR5 in whole cell extract of LM7 cells by western blot. Whole cell extract from U87 cells was used as a positive control for mGluR5 expression. We observed mGluR5 expression in LM7 cells (Fig 5E), however, we did not detect mGluR1 protein expression (data not shown). These data indicated that of the Type 1 receptors, mGluR5 is predominantly expressed in LM7 cells. Specifically, blocking the activation of mGluR5 by Fenobam significantly reduced total number of cells, proliferating cells, and induced apoptosis, which is similar to the effect of Riluzole on LM7 cells. These data suggests that mGluR5 plays a key role in the proliferation of LM7 cells.

### Riluzole alters phosphorylation status of downstream signaling molecules

**Fig 6:**
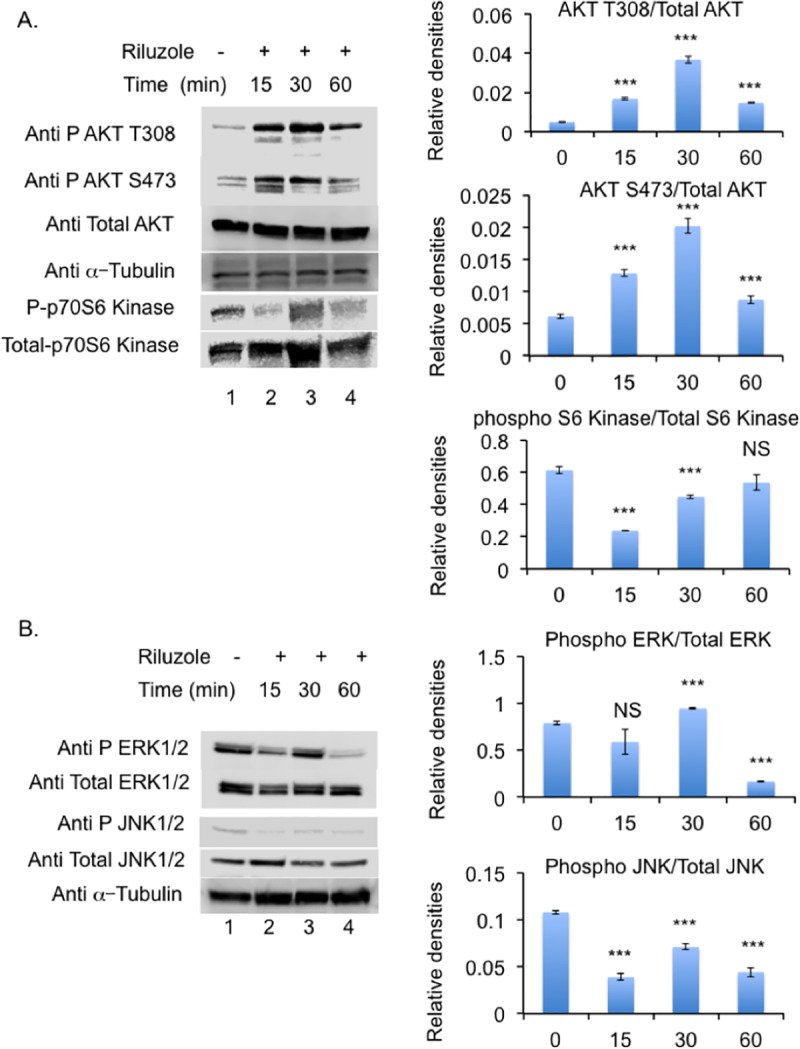
Riluzole alters phosphorylation status of AKT, ERK1/2 and JNK. **A.** Western blotting was carried out using antibodies against Phospho AKT T308 or phospho AKT S473 or total AKT or phospho P70S6 Kinase or total P70S6 kinase. Relative densities of the western blots are shown in the right panel. P value<0.0001 = *** and NS = not significant. **B** Antibodies against Phospho ERK1/2, total ERK1/2, Phospho JNK1/2, and total JNK1/2 were tested. Tubulin was used as a loading control. Relative densities of the western blots are shown in the right panel. P value<0.0001 = *** and NS = not significant.

mGluR1/5 are known to signal through PI3 Kinase which are tethered to the membrane via Homer to initiate signaling in metabolism, cell survival, proliferation and tumor progression [36, 37]. To further determine the effect of Riluzole on glutamate induced signaling pathways we examined the phosphorylation status of signaling proteins such as Akt, which participates in signaling survival, cell growth, cell proliferation, and migration in normal and cancer cells [38, 39]. Riluzole treatment induced robust increase in phosphorylation at AKT Threonine 308 at 15 with peak at 30 minutes. Similarly, Riluzole increased phosphorylation at AKT Serine 473 with a peak at 15 and 30 minutes. (Fig 6A). Total Akt was used to obtain relative densities of the bands at different time points and μ-Tubulin used as a loading control shows equal protein in all lanes. Increase in phosphorylation at T308 or S473 was highly significant with a p value of < 0–0001. Akt phosphorylates mTOR, which is involved in cell proliferation and cell survival. Phosphorylation of P70 S6 Kinase at threonine 389 is a hallmark of mTOR activation [40]. Therefore, we also examined the phosphorylation status of P70 S6 Kinase after Riluzole treatment and observed decrease in phosphorylation at 15, 30 minutes, however, at 60 the phosphorylation was similar to that of the control. The decrease in phosphorylation at 15 and 30 minutes was highly significant with a p value of <0.0001 (Fig 6A). The phsophorylation of ERK1/2 showed an increase at 30 minutes and decreased at 60 minutes after treatment with Riluzole which was highly significant with a p value of <0.0001. Riluzole also altered the phsophorylation status of JNK1/2. Riluzole decreased the phosphorylation of JNK1/2 at 15 minutes, 30 minutes and 60 minutes after treatment, the difference was highly significant compared to untreated control with a p value of <0.0001. Total ERK1/2 and total JNK1/2 were used to obtain the relative densities of the phosphorylated protein at each time point (Fig 6B). Hence, Riluzole treatment alters phosphorylation status of AKT at T308 and S473, P70 S6 kinase, ERK1/2 and JNK1/2. Thus Riluzole affects signaling pathways involved in cell survival, cell growth and proliferation.

### Knockdown of mGluR5 leads to inhibition of growth

**Fig 7:**
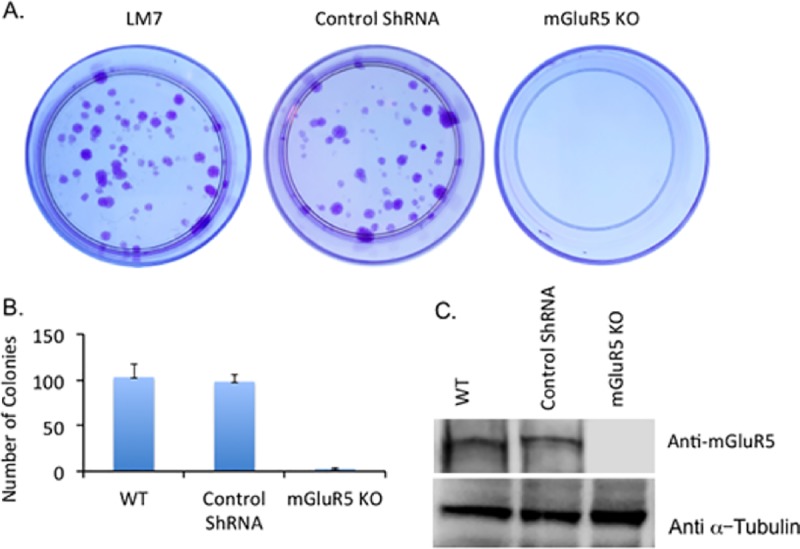
Knockdown of mGluR5 slows growth of LM7 cells. Colonies stained by Cresyl Violet are shown from WT, control ShRNA and mGluR5 KO samples. **B.** The number of colonies in each sample from three independent experiments. **C.** Western blot of whole cells extracts from WT, control ShRNA and mGluR5 KO using anti mGluR5 antibody. μ-Tubulin is used as a loading control.

**Fig 8:**
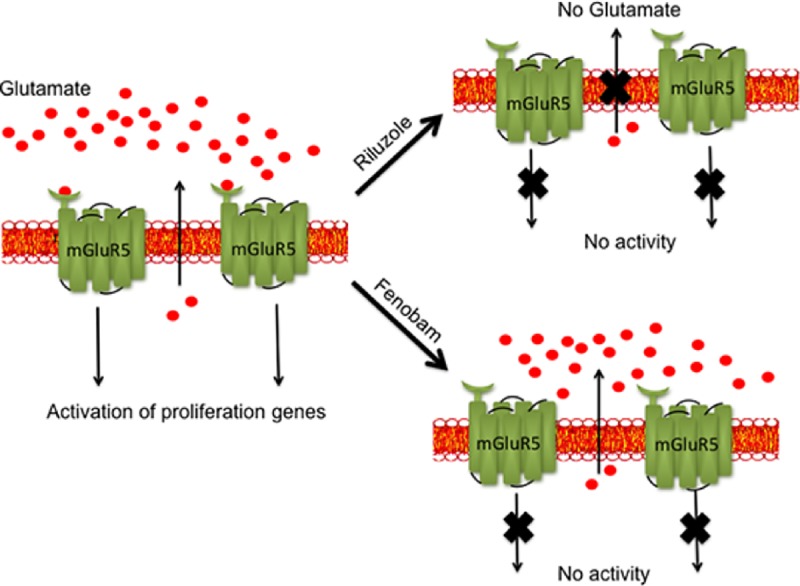
Schematic of mechanism of action of Riluzole. Glutamate released from LM7 cells binds and activates mGluR receptor signaling that in turn induces the activation of genes involved in proliferation. Riluzole treatment inhibits glutamate release thereby preventing the activation of glutamate receptors. Similarly, blocking of mGluR receptors with Fenobam also prevents mGluR5 activation and signaling thereby blocking activation of proliferation genes.

To further verify that mGluR5 is the key receptor involved in glutamate-dependent proliferation of LM7 cells, we knocked down the expression of mGluR5 using Lentivirus expressing ShRNA for mGluR5. The stable cell lines expressing ShRNA for mGluR5 or non-target ShRNA were selected using puromycin in the culture media. A colony-forming assay was performed as described in Materials and Methods. Wild type LM7 and non-target ShRNA control show visible colonies after two weeks incubation. However, mGluR5 KO cells did not show visible colonies (Fig 7A). The number of colonies in the plates were quantified Fig 7B. Expression of mGluR5 was determined using western blot of whole cell extracts using anti-mGluR5 antibody (Fig 7C). mGluR5 KO does not show expression of mGluR5 while WT and control ShRNA samples show mGluR5 expression. The results provide evidence that mGluR5 plays an important role in signaling glutamate dependent proliferation in LM7 cells. The schematic of inhibition of LM7 cell proliferation by Riluzole or Fenobam is depicted (Fig 8).

## Discussion

In the present study we have demonstrated that Riluzole inhibits proliferation, induces apoptosis of human osteosarcoma, LM7 and mouse osteosarcoma OS482 cells. Riluzole also prevents migration in LM7, osteosarcoma cells. Loss of migration in Riluzole treated cells could be largely due to these cells undergoing apoptosis. We have also shown that blocking mGluR5 receptor activation by allosteric modulator, Fenobam, prevented proliferation and induced apoptosis. Knock down of mGluR5 resulted in significant decrease in colony forming ability of the LM7 cells and mimics the effects of Riluzole treatment. We further demonstrate that signaling through mGluR5 via the AKT pathway is altered along with ERK1/2 and JNK1/2 pathway. Thus Riluzole likely prevents the activation of mGluR5 receptor and other plausible receptors or channels that may be involved in proliferation to exert its effects on osteosarcoma cells. Riluzole has been employed successfully to target melanoma cells, triple negative breast cancer cells and has been tested in clinical trial for melanoma [24, 28, 29, 41]. Riluzole is used for treating Amyotropic Lateral Sclerosis patients for over two decades and has been shown to reduce glutamate levels in the cerebrospinal fluid and prolong life span [42–44]. Riluzole is an approved drug by FDA, its toxicity is characterized and is well tolerated in patients [45]. We therefore investigated the use of Riluzole and an anti-cancer agent for osteosarcoma using LM7 cells.

Glutamate signaling was first demonstrated in osteoblasts and osteoclasts as part of normal bone signaling [46, 47]. Spontaneous secretion of glutamate by osteosarcoma cells was demonstrated in MG63 and Saos-2 cells lines. However, osteocyte like MLO-Y4 cells did not release glutamate [48]. MG63 cells were later shown to express various types of glutamate receptors using RT-PCR [49]. Although glutamate signaling is important in normal bone growth and remodeling, a genome wide association study, using 941 osteosarcoma patients samples and 3291 cancer free adults of European ancestry, concluded that mGulR4 loci is affected in osteosarcoma. A later study using osteosarcoma samples from 40 patients and 118 paraffin embedded osteosarcoma tissues showed that osteosarcoma samples express higher levels of mGluR4 compared to normal bone tissue [50]. The same study also showed higher expression of mGluR1 and mGluR5 in osteosarcoma [50]. In osteoblast-like osteosarcoma cells, MG63 cells dexamthasone induced expression of mGluR5 [51]. Interestingly, a recent study has shown that only 20% of osteosarcoma screened showed higher mGluR4 expression [52]. Exaggerated glutamate signaling plays an important role in tumorigenicity and malignancy of osteosarcoma.

We have demonstrated that LM7 cells secrete glutamate and blocking glutamate secretion with Riluzole inhibited their growth. The viability of cells in all samples for glutamate assay remained high around 98% ensuring that the glutamate released was indeed from live cells and not from lysed dead cells. Riluzole also decreased Ki-67 positive cells indicative of loss of proliferating cells. Importantly, in bone tumors Ki-67 expression level is correlated with degree of malignancy and serves well in prognosis [53]. It is conceivable that Riluzole might be targeting proliferating cells to induce apoptosis. Apoptosis induced by Riluzole suggests the importance of autocrine signaling of glutamate in LM7 cells. LM7 cells are adapted to exploit glutamate signaling for their growth and preventing glutamate release results in apoptosis. We have observed a marginal increase in growth by DHPG, which is most likely due to saturation of receptors due excessive levels of glutamate. DHPG was unable to rescue inhibition by Riluzole or Fenobam suggesting that these two drugs showed a strong inhibition of mGluR5 receptor, which could not be reversed. In contrast, CPCCOEt did show significant inhibition of cell proliferation (p value <0.01), however, CPCCOEt did not induce significant apoptotic death suggesting that inhibition of mGluR1 receptor activity altered proliferation status of the cells but did not induce apoptosis in the cells. Therefore, mGluR1 may not be playing a major role in survival of LM7 cells. This was further corroborated by the lack of mGluR1 protein expression. It is interesting to note that in breast cancer, Riluzole was shown to exert anti-tumorigenic activity independent of mGluR1 expression [54]. Furthermore, Riluzole induced apoptosis in prostrate cancer through endoplasmic stress [27].

In the present study we also demonstrated that AKT phosphorylation at T308 and S473 is altered upon Riluzole treatment. Riluzole treatment also altered p70 S6 kinase phosphorylation demonstrating that mGluR5 signaling through PI3/AKT/mTOR pathway is affected. It is well known that mGluR1/5 receptors are linked to and activate the PI3/AKT/mTOR pathway [19, 55]. Our data showed that Riluzole also altered phosphorylation status of ERK1/2. Interestingly, mGluR5 has been shown to activate ERK1/2 signaling via G-alpha q-protein but not through phospholipase C beta1 [56]. We did not observe any changes in PLCμ phosphorylation status upon Riluzole treatment (data not shown). Furthermore, we show Riluzole treatment results in altered phosphorylation of JNK1/2. Signaling through G-alpha q-protein coupled mGluR5 receptors results in transactivation of EGF receptor, which in turn activates JNK phosphorylation [57, 58]. Although mGluR5 activation can lead to changes in more than one signaling pathway, it is conceivable that Riluzole could be blocking other glutamate receptors that may be present in LM7 cells. We have tested for the expression of AMPA receptors subunits GluR1, GluR2 and NMDA receptor subunits NR1, NR2A, NR2B and did not detect their expression in LM7 cells (data not shown). However, we have not tested for the expression of mGluR4 in LM7 cells, which is known to activate cAMP pathway. Knock down of mGluR5 resulted in significant decrease in colony formation ability of mGluR5 KO LM7 cells. The mGluR5 knock down data along with inhibitor data suggests mGluR5 is indeed a key player in signaling growth in LM7 cells.

In conclusion, we have demonstrated that treatment with Riluzole or blocking mGluR5 receptors with Fenobam decreases cell proliferation and induces apoptosis in LM7 cells suggesting that mGluR5 receptors might play a key role in the proliferation of LM7 cells. Moreover, knock down of mGluR5 receptors significantly slowed proliferation of LM7 cells. Riluzole disrupts autocrine signaling by depriving the cells of extracellular glutamate preventing activation of mGluR5. Riluzole blocks glutamate secretion, however, Fenobam prevents activation of mGluR5 receptor both Riluzole and Fenobam prevent proliferation of LM7 cells as depicted in Fig 8. Notably, Riluzole also blocked proliferation and induced apoptosis in OS482 cells. Therefore, treatment with Riluzole may be effective in inhibiting growth and inducing apoptosis in osteosarcoma cells that utilize glutamate for growth signaling. Our data provides evidence for the therapeutic potential of Riluzole in osteosarcoma.
